# ARID1A facilitates KRAS signaling-regulated enhancer activity in an AP1-dependent manner in colorectal cancer cells

**DOI:** 10.1186/s13148-019-0690-5

**Published:** 2019-06-19

**Authors:** Madhobi Sen, Xin Wang, Feda H. Hamdan, Jacobe Rapp, Jessica Eggert, Robyn Laura Kosinsky, Florian Wegwitz, Ana Patricia Kutschat, Fereshteh S. Younesi, Jochen Gaedcke, Marian Grade, Elisabeth Hessmann, Argyris Papantonis, Philipp Strӧbel, Steven A. Johnsen

**Affiliations:** 1 Department of General, Visceral and Pediatric Surgery, University Medical Center Göttingen, 37075 Göttingen, Germany; 2 Gene Regulatory Mechanisms and Molecular Epigenetics Lab, Gastroenterology Research, Mayo Clinic, 200 First Street SW, Rochester, MN 55905 USA; 3 Department of Gastroenterology & Gastrointestinal Oncology, University Medical Center Gӧttingen, 37075 Göttingen, Germany; 4 Department of Pathology, University Medical Center Göttingen, 37075 Göttingen, Germany

**Keywords:** ARID1A, BAF complex, *KRAS*, MEK/ERK pathway, AP1, Enhancers, Transcriptional regulation, Colorectal cancer

## Abstract

**Background:**

ARID1A (AT-rich interactive domain-containing protein 1A) is a subunit of the BAF chromatin remodeling complex and plays roles in transcriptional regulation and DNA damage response. Mutations in *ARID1A* that lead to inactivation or loss of expression are frequent and widespread across many cancer types including colorectal cancer (CRC). A tumor suppressor role of ARID1A has been established in a number of tumor types including CRC where the genetic inactivation of *Arid1a* alone led to the formation of invasive colorectal adenocarcinomas in mice. Mechanistically, ARID1A has been described to largely function through the regulation of enhancer activity.

**Methods:**

To mimic ARID1A-deficient colorectal cancer, we used CRISPR/Cas9-mediated gene editing to inactivate the *ARID1A* gene in established colorectal cancer cell lines. We integrated gene expression analyses with genome-wide ARID1A occupancy and epigenomic mapping data to decipher ARID1A-dependent transcriptional regulatory mechanisms.

**Results:**

Interestingly, we found that CRC cell lines harboring *KRAS* mutations are critically dependent on ARID1A function. In the absence of ARID1A, proliferation of these cell lines is severely impaired, suggesting an essential role for ARID1A in this context. Mechanistically, we showed that ARID1A acts as a co-factor at enhancers occupied by AP1 transcription factors acting downstream of the MEK/ERK pathway. Consistently, loss of *ARID1A* led to a disruption of KRAS/AP1-dependent enhancer activity, accompanied by a downregulation of expression of the associated target genes.

**Conclusions:**

We identify a previously unknown context-dependent tumor-supporting function of ARID1A in CRC downstream of KRAS signaling. Upon the loss of ARID1A in *KRAS*-mutated cells, enhancers that are co-occupied by ARID1A and the AP1 transcription factors become inactive, thereby leading to decreased target gene expression. Thus, targeting of the BAF complex in *KRAS*-mutated CRC may offer a unique, previously unknown, context-dependent therapeutic option in CRC.

**Electronic supplementary material:**

The online version of this article (10.1186/s13148-019-0690-5) contains supplementary material, which is available to authorized users.

## Background

In 2018, colorectal cancer (CRC) was estimated to be the third most commonly occurring cancer and the second leading cause of cancer-related deaths [1]. An accumulation of mutations in several key pathways occurs during the transformation from normal colonic epithelium to a malignant carcinoma [2]. Genes involved in these pathways such as *APC*, *KRAS*, and *TP53* represent a large fraction of the mutations prevalent in CRC. Notably, the *KRAS* oncogene is mutated in approximately 30% of CRC cases (cBioPortal for Cancer Genomics) [3, 4]. These mutations generally lead to increased proliferation and survival via downstream activation of signaling through the Raf/MEK/ERK cascade [5]. KRAS signaling ultimately relays information to the intracellular transcriptional machinery via AP1 transcription factors, which dimerize and bind to the DNA upon phosphorylation, where they recruit further transcriptional regulators to modulate gene expression [6, 7].

Followed by mutation of well-known tumor suppressor and oncogenes such as those listed above, the *ARID1A* gene is among the most frequently mutated genes in human CRC, where it is mutated in 10.9% of cases (TCGA PanCancer Atlas dataset, cBioPortal for Cancer Genomics) [3, 4]. Interestingly, this frequency is even higher than mutational rates of several other bona fide tumor suppressor genes such as *PTEN* (6.4%) (cBioportal for Cancer Genomics) [3, 4]. ARID1A is a subunit of the human BAF (BRG1-associated factors) complex [8, 9], which is primarily involved in chromatin remodeling. Chromatin remodelers such as the BAF complex are large, multi-subunit complexes that utilize the energy of ATP hydrolysis to mobilize, slide, and evict nucleosomes [10, 11]. In vitro, four core subunits are required to dissociate nucleosomes from the DNA on a chromatin template. These include the mutually exclusive ATPases SMARCA2 or SMARCA4 (BRG1) and core subunits SMARCB1, SMARCC1, and SMARCC2 that enhance catalytic activity [12]. The exact role of the other BAF subunits is not very well understood, but mutation rates in cancer suggest important roles in vivo. As a regulator of chromatin structure and function, the BAF complex plays crucial roles in transcription and epigenetic modulation of gene expression [13–17].

The deregulation of epigenetic modulation has been well established as a common occurrence in cancer. However, the extent of its involvement in the development and progression of cancer was underscored by genome- and exome-wide sequencing studies which revealed a close association between the epigenome and the pathogenesis of cancer. Most significantly, subunits of the mammalian BAF complex show an alteration frequency in over 20% of all cancers [18, 19]. Among BAF complex subunits, *ARID1A* mutations are the most recurrent and widespread across many cancer types [18]. These mutations often lead to a loss of ARID1A expression in tumors, and *ARID1A* has been extensively described as a tumor suppressor in the literature [20–25]. Consistently, the sporadic deletion of *Arid1a* in mice led to the formation of invasive adenocarcinomas in the colon [21].

While the BAF complex has also been shown to play a role in DNA damage repair [26], its primary role appears to be in enhancer-mediated gene regulation [21, 27–32]. Enhancers are transcriptionally active, distal regulatory elements which modulate the expression of their target genes [33]. While initial studies reported that ARID1A occupied promoter regions [34, 35], several studies have since shown that the BAF complex is targeted to enhancers [21, 27–32]. In fact, the tumor suppressive functions of the BAF complex have been suggested to be primarily through its function in controlling enhancer-mediated gene regulation [21, 27, 29]. In colorectal cancer, it was shown that the activity of BAF-occupied enhancers is reduced in *ARID1A*-deficient cells and accompanied by a loss of the active H3K27ac mark (acetylation of lysine 27 on histone 3) [21]. In this study, we sought to examine the transcriptional mechanisms underlying the tumor suppressor function of ARID1A in colorectal cancer. Surprisingly, we uncovered a previously unknown context-dependent function of ARID1A in promoting AP1 transcription factor activity downstream of KRAS signaling.

## Results

### ARID1A protein loss is a frequent event in colorectal cancer

**Fig. 1:**
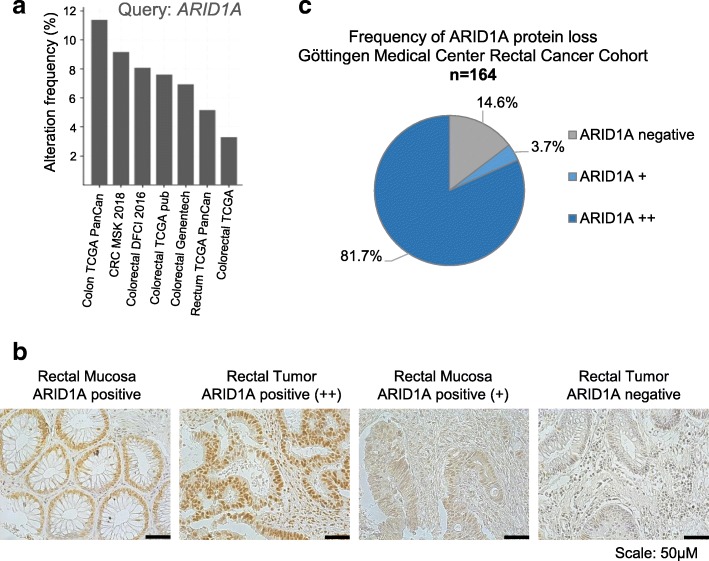
ARID1A protein loss in human cancer samples is consistent with mutation rates. *ARID1A* is mutated in up to ~ 12% colorectal cancers from different colorectal cancer cohorts [3, 4] (**a**). Representative immunohistochemical staining images show clear ARID1A staining in the rectal mucosa and positive tumors while negative tumors show no staining at all (**b**). The University Medical Center Gӧttingen rectal cancer cohort showed 81.7% strongly positive ARID1A staining (++) and 3.7% showed weakly positive staining (+) while 14.6 % of the samples were negative for ARID1A staining (**c**)

To begin with, we examined the mutation frequency for *ARID1A* in colorectal cancer patients. Analysis of several large datasets revealed an alteration frequency of up to ~ 12% (cBioPortal for Cancer Genomics) [3, 4] (Fig. 1a). Since mutations in *ARID1A* have been shown to lead to the loss of protein expression in tumors [25, 36–38], we next examined the frequency of the loss of ARID1A protein expression by immunohistochemical analysis of tissue microarrays from treatment naïve rectal cancer patients. As shown in the representative images in Fig. 1b, the immunohistochemical staining for ARID1A was specific with normal rectal mucosa demonstrating positive ARID1A staining, whereas tumors were either strongly positive (++), weakly positive (+), or negative for ARID1A expression (negative). Overall, out of 164 patients, 81.7% retained ARID1A expression in their tumors (Fig. 1c). Consistent with the mutation rates, we observed a complete loss of ARID1A protein expression in 14.6% of the cases (Fig. 1c) and 3.7% of patient tumors showed a weak staining pattern (Fig. 1c). The weak positivity could perhaps be attributed to a heterozygous loss of *ARID1A*. These findings demonstrate that the loss of ARID1A protein expression is a frequent occurrence in rectal cancer patients.

### The loss of *ARID1A* leads to impaired proliferation in a subset of colorectal cancer cell lines

**Fig. 2:**
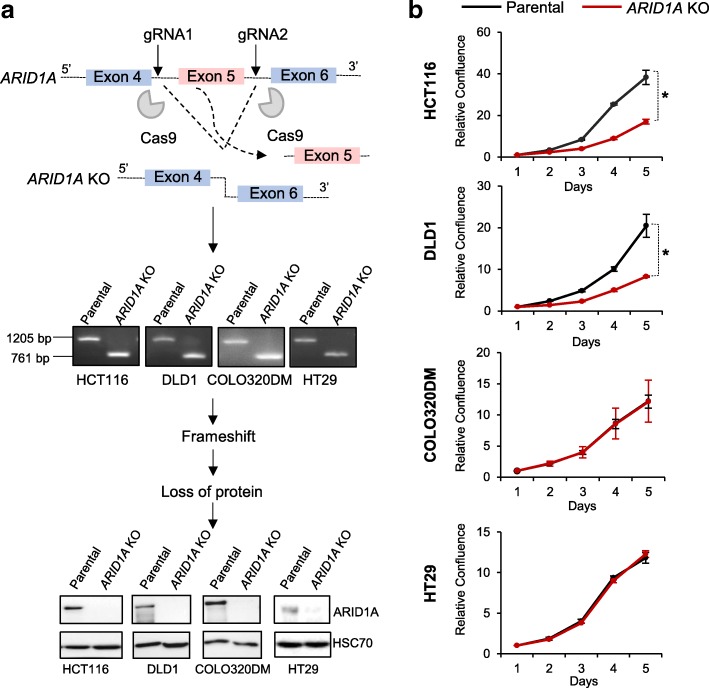
The loss of ARID1A impairs proliferation of a subset of colorectal cancer cell lines. To mimic *ARID1A*-deficient colorectal cancer, we used CRISPR/Cas9-mediated gene editing to delete exon 5 of the *ARID1A* gene from four colorectal cancer cell lines HCT116, DLD1, COLO320DM, and HT29. Regions flanking exon 5 were targeted by two guide RNAs leading to an out of frame gene product. Genotyping PCRs show a shorter product of 761 bp in the *ARID1A* knockout (KO) cells (**a**, middle panel). This led to a complete loss of the ARID1A protein from the KO cells (**a**, lower panel). HSC70 is used as a loading control. Representative graphs show that the proliferation of the HCT116 and DLD1 cell lines is significantly impaired by the deletion of *ARID1A*, whereas the proliferation of COLO320DM and HT29 is unaffected by this knockout (**b**). Error bars represent standard deviation between the replicates. *n* for proliferation assay = 2, *p < 0.05 unpaired *t* test

Given its pivotal tumor suppressive role in vivo, we next sought to explore the consequences of *ARID1A* loss at the molecular level in an in vitro system. For this purpose, we utilized a CRISPR/Cas9-mediated gene editing approach to delete a portion of the *ARID1A* gene in four colorectal cancer cell lines (COLO320DM, DLD1, HCT116, and HT29). These cell lines were specifically chosen because they expressed several BAF complex subunits to varying degrees (Additional file 1: Figure S1a) and also represent a varied mutational background representative of the diversity found in human CRC tumors. For example, HCT116 and DLD1 both contain *KRAS*^G13D^ mutations, whereas COLO320DM and HT29 are wild type for this gene. Similarly, all cell lines except HCT116 harbor mutations in *TP53* (cBioPortal for Cancer Genomics) [3, 4]*.* The guide RNAs utilized specifically target two intronic regions flanking exon 5 of *ARID1A*, resulting in a frameshift of any potentially transcribed and spliced product from the mutant allele (Fig. 2a). We screened single cell clones of each cell line for deletion (KO) of *ARID1A* by genotyping PCR (Fig. 2a) and Western blot for protein (Fig. 2a). Genotyping PCR with primers targeting regions flanking the deleted exon revealed a product of 761 bp in the knockout cells as compared to 1205-bp in the parental condition. The production of an out of frame product led to the complete loss of the protein from the cells, as can be seen for all four cell lines in Fig. 2a. Thus, we were able to produce cellular model systems to study the functional consequences of ARID1A loss in the context of other commonly occurring mutations.

After generating the *ARID1A*-deficient cell lines, we next sought to characterize them phenotypically. Surprisingly, in contrast to its described tumor suppressive role, we observed that the deletion of *ARID1A* led to a severe impairment in proliferation in two cell lines (HCT116 and DLD1) while proliferation in the other two cell lines (COLO320DM and HT29) was unaffected (Fig. 2b). Thus, loss of *ARID1A* differentially affects colorectal cancer cell proliferation.

### ARID1A is required for MEK/ERK pathway-induced transcription

**Fig. 3:**
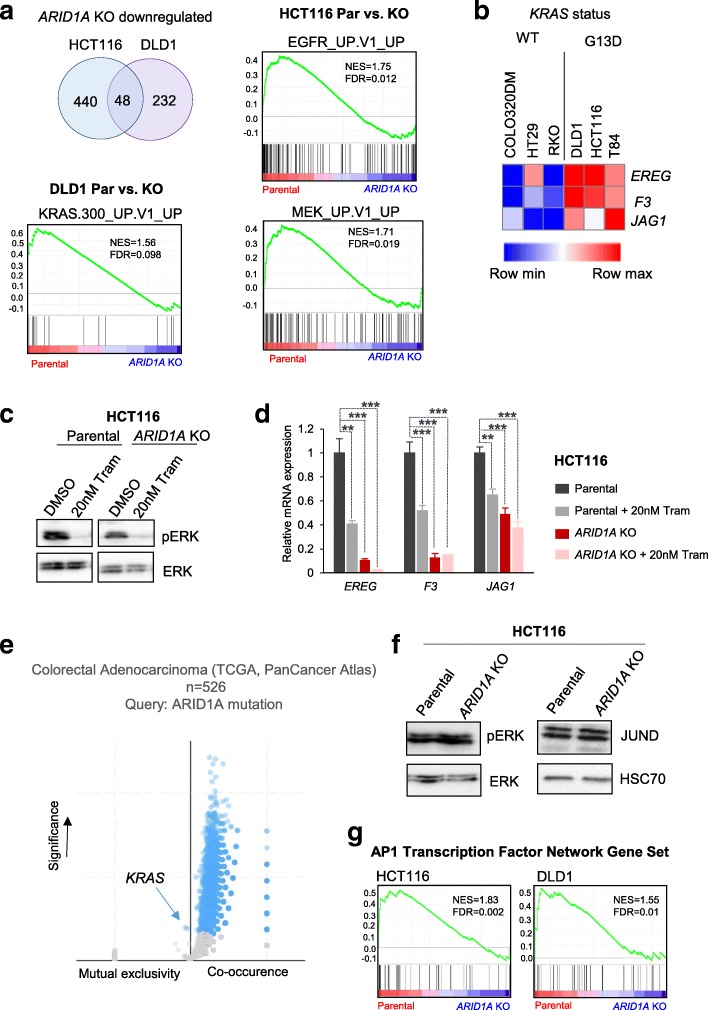
Loss of *ARID1A* results in deregulated expression of MEK/ERK pathway target genes. Forty-eight genes are commonly downregulated in the HCT116 and DLD1 cell lines (**a**). Gene set enrichment analysis shows that gene sets containing targets of the MEK/ERK pathway are enriched in the parental condition (**a**). The genes *EREG*, *F3*, and *JAG1* are highly expressed in cell lines with *KRAS*^G13D^ mutations as analyzed using on the Morpheus tool to analyze data from the Cancer Cell Line Encyclopedia (CCLE) [36] (**b**). The scale represents the minimum expression in a particular row (blue) to the maximum expression in that row (red). Treatment with 20 nM trametinib for 24 h leads to a reduction of phosphorylated ERK (pERK) (**c**). The expression of *EREG*, *F3*, and *JAG1* was significantly reduced by trametinib treatment and *ARID1A* deletion. Treatment with trametinib in *ARID1A*-deficient cells did not lead to a further reduction of gene expression (**d**). *ARID1A* mutations are significantly mutually exclusive with *KRAS* mutations in the TCGA colorectal cancer patient cohort (**e**). The *y*-axis represents −log10 (*p* value) and the blue dots represent significantly mutually exclusive or co-occurring mutations. pERK and JUND levels remain unchanged in *ARID1A*-deficient cells. HSC70 is used as a loading control. The AP1 transcription network gene set [41] is enriched in the parental condition (**f**). qRT-PCRs were performed for biological triplicates and technical duplicates. Error bars represent the standard deviation between three biological replicates. Significance was calculated using an unpaired *t* test, *p < 0.05, **p < 0.01, ***p < 0.001

To identify the gene regulatory networks responsible for the observed proliferation defects, we performed analyses of mRNA-sequencing data in HCT116 and DLD1 parental and *ARID1A*-deleted cell lines. Since the BAF complex has primarily been described to be a transcriptional activator and function in enhancer activation, we focused on genes that were downregulated upon ARID1A loss to identify potential direct transcriptional targets. Pathway enrichment analysis for the set of downregulated genes in each of these cell lines (Additional file 1: Figure S1d) revealed considerable variability between the three cell lines. Notably, the two cell lines displaying proliferation defects (HCT116 and DLD1) showed an overlap of 48 downregulated genes upon *ARID1A* KO (Fig. 3a). While in the HCT116 cell line several transcriptional regulatory pathways were affected, COLO320DM cells (whose proliferation was unaffected by *ARID1A* loss) displayed changes in gene expression associated with cardiac differentiation and Wnt pathway.

Further gene set enrichment analyses (GSEA) revealed that genes controlled by MEK/ERK pathway components (EGFR, MEK, and ERK) were specifically downregulated following ARID1A loss in HCT116 (EGFR, MEK) and DLD1 (KRAS) cells (Fig. 3a). This finding is consistent with these cell lines both containing mutations in the *KRAS* gene. To further investigate whether ARID1A may preferentially promote the expression of downstream KRAS target genes, we compared three *KRAS*-mutated cell lines (DLD1, HCT116, T84) to three wild-type counterparts (COLO320DM, HT29, RKO) using the Morpheus tool [39]. We identified *EREG*, *F3*, and *JAG1* as potential candidates that were particularly highly expressed in the *KRAS*-mutated cell lines compared to cell lines harboring wild-type *KRAS* (Fig. 3b). Notably, the three *KRAS*-mutated CRC cell lines all harbored activating mutations at residues G12 or G13. While not previously characterized as typical downstream MEK/ERK targets, each of these genes has potential interesting roles in the development of cancer. EREG (Epiregulin) is a ligand for EGFR (epidermal growth factor receptor) which signals through the MEK/ERK pathway [40]. One study suggested that low EREG expression is associated with better overall survival in colorectal cancer patients [41]. *F3* (tissue factor III) encodes a glycoprotein receptor for coagulation factor VII and initiates blood coagulation, but has also been implicated in cancer metastasis [42]. JAG1 (Jagged1) is a ligand for the Notch receptor, and high expression has been associated with poor prognosis in CRC [43]. To test whether these genes are, in fact, targets of the MEK/ERK pathway, we inhibited the pathway by treating cells with the clinical MEK1/2 inhibitor trametinib. Treatment with 20 nM trametinib for 24 h reduced the levels of phosphorylated ERK completely (Fig. 3c), thus blocking the activation of downstream targets of this pathway. Consistent with their co-expression in *KRAS*-mutated CRC cell lines, *EREG*, *F3*, and *JAG1* were all downregulated following trametinib treatment, thereby confirming that they are, indeed, MEK/ERK target genes (Fig. 3d). Importantly, consistent with our GSEA analyses, all three investigated MEK/ERK target genes were also downregulated by the loss of ARID1A. Furthermore, consistent with ARID1A functioning epistatically downstream of the KRAS-dependent MEK/ERK signaling, trametinib did not substantially further reduce the expression of these genes in the absence of ARID1A expression. Therefore, our results suggest that ARID1A is required for maintaining the expression of cancer-relevant downstream targets of the MEK/ERK pathway in *KRAS*-mutated CRC cells.

### ARID1A is essential in *KRAS*-mutated colorectal cancer cells and controls the MEK/ERK pathway at the transcriptional level

Based on our results above, indicating a positive role for ARID1A downstream of the MEK/ERK pathway, we reasoned that mutations of *ARID1A* and *KRAS* would not be compatible since a loss of ARID1A would impede the effects of hyperactive MEK/ERK signaling. Indeed, examination of the TCGA PanCancer Atlas colorectal adenocarcinoma cohort revealed that *KRAS* mutations were mutually exclusive with *ARID1A* mutations with only 16 out 526 patients harboring mutations in both genes (Fig. 3e, Additional file 1: Figure S1b). This mutual exclusivity was even more pronounced when the analysis was restricted to *KRAS* mutations in the codons for residues G12 and G13 (Additional file 1: Figure S1b). Interestingly, the mutation rates of 17% for *ARID1A* are significantly higher in *KRAS*-wildtype tumors than in *KRAS*-G12/G13-mutated samples. This finding is consistent with our in vitro findings that the *KRAS*-mutant colorectal cancer cells HCT116 and DLD1 (KRAS^G13D^ mutation) displayed proliferation defects (Fig. 2b) upon the loss of ARID1A. Therefore, in the context of *KRAS* mutations, ARID1A does not appear to function tumor suppressive, but rather facilitates the expression of genes particularly dependent on the MEK/ERK pathway.

To further explore ARID1A function in the *KRAS*-mutant context, we investigated whether ARID1A loss directly regulated MEK/ERK pathway signaling activity by examining the levels of phosphorylated ERK (pERK) in wild-type and *ARID1A*-deficient HCT116 cells. However, loss of ARID1A expression did not result in decreased phosphorylation of ERK in either HCT116 (Fig. 3f) or DLD1 cells (Additional file 1: Figure S1c). Moreover, the expression of one of the most highly expressed AP1 factors, JUND, was also unaffected by *ARID1A* loss in either HCT116 (Fig. 3f) or DLD1 cells (Additional file 1: Figure S1c), suggesting that the observed perturbation of downstream MEK/ERK target genes was not due to altered expression of JUND. We therefore reasoned that ARID1A may control the transcriptional activation capacity of AP1 transcription factors and tested this hypothesis by performing GSEA using a set of genes associated with the AP1 transcription network [44]. Consistent with our hypothesis, this gene set was specifically downregulated in *ARID1A*-deficient HCT116 and DLD1 cells in comparison with the parental cell lines (Fig. 3g). Thus, together, these findings suggest that ARID1A regulates target gene expression of the MEK/ERK pathway directly at the transcriptional level without appreciably affecting upstream signaling.

### ARID1A localizes to AP1-bound enhancer regions

**Fig. 4:**
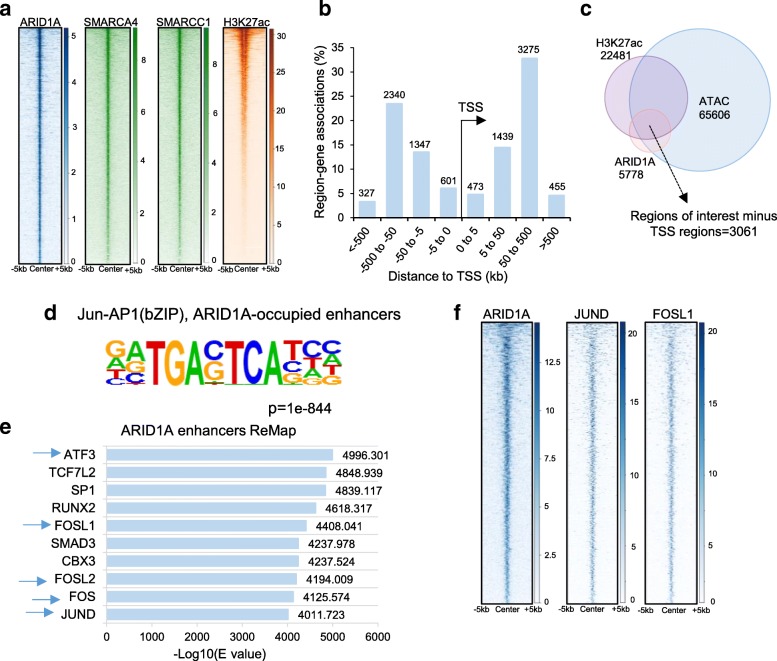
ARID1A is localized to AP1-occupied enhancers. ARID1A co-localizes with the BAF complex subunits SMARCA4 and SMARCC1 as well as the active mark H3K27ac. The heatmaps represent all ARID1A binding sites ordered in descending order of ARID1A occupancy (**a**). ARID1A localizes mainly to regions that are distal to transcription start sites (TSS) with the maximum number of peaks between 5 and 500 kb of TSSs (**b**). ARID1A-bound enhancers were defined based on overlap of ARID1A with H3K27ac and ATAC-seq. From these regions, any annotated TSSs were subtracted and 3061 ARID1A-bound enhancers were identified (**c**). Motif analysis on ARID1A-bound enhancers shows enrichment for the Jun-AP1(bZIP) motif (**d**). ReMap analysis shows that several AP1 transcription factors such as FOSL1, FOSL2, FOS, and JUND are enriched on ARID1A-occupied regions identified in HCT116 cells (**e**). Co-localization of JUND and FOSL1 at ARID1A-bound enhancers in HCT116 cells is shown. The heatmaps represent the 3061 ARID1A-bound enhancers in descending order of ARID1A occupancy (**f**)

We next sought to examine the direct roles of ARID1A in controlling transcription downstream of the MEK/ERK pathway by examining the genome-wide occupancy of ARID1A. To date, most conclusions about ARID1A function in transcription have been through indirect information based on SMARCA4 and SMARCC1 occupancy. Thus, in order to gain further direct, causal insights, we established chromatin immunoprecipitation for ARID1A utilizing a recently established dual crosslinking approach [45]. This approach yielded good quality ChIP-seq data, enabling the direct examination of ARID1A occupancy. Bioinformatic analysis of these data confirmed a strong overlap with the BAF complex subunits SMARCA4 and SMARCC1 and co-occupancy with H3K27ac, which specifically marks active enhancer and promoter regions (Fig. 4a). Furthermore, consistent with recent literature demonstrating the importance of the BAF complex in enhancer-mediated regulation, the majority of ARID1A-enriched regions were distal (5 to > 500 kb) to the nearest transcriptional start site (TSS) (Fig. 4b). To further examine ARID1A function at enhancer regions, we specifically identified active, ARID1A-bound regions which were enriched for H3K27ac and contained accessible chromatin regions (based on ATAC-seq) (Fig. 4c). To specifically examine ARID1A function at enhancers, we further subtracted any regions that were annotated as transcriptional start sites, yielding 3,061 potential ARID1A-bound enhancer regions. Subsequently, we performed sequence-based motif analyses on these regions to identify enriched DNA-binding sequences indicative of specific transcription factor dependencies. Consistent with our hypothesis and previous studies examining BAF occupancy in other systems [21, 28], we observed that ARID1A-bound enhancers are particularly enriched in AP1 binding motifs (Fig. 4d). This finding was confirmed by comparing the ARID1A-bound regions with publicly available ChIP-seq data using the ReMap tool, where AP1 factors were among the top hits that co-localize with ARID1A (Fig. 4e). Importantly, among the overlapping factors were FOSL1 and JUND, which are among the most highly expressed AP1 factors in HCT116 cells. We confirmed these findings using ChIP-seq data for FOSL1 and JUND occupancy in HCT116 cells, where we observed a strong co-occupancy of FOSL1 and JUND at the 3,061 ARID1A-bound enhancer regions (Fig. 4f). Motif analysis and ReMap-based transcription factor occupancy for all ARID1A binding sites are shown in Additional file 1: Figure S2a, b. Thus, in conclusion, global ChIP-seq analysis revealed that ARID1A co-localizes with AP1 factors, thereby supporting a potential direct role of ARID1A in directing AP1-dependent enhancer-mediated gene regulation downstream of the MEK/ERK pathway.

### The loss of ARID1A leads to a significant reduction of H3K27ac at enhancers and downregulation of target genes

**Fig. 5:**
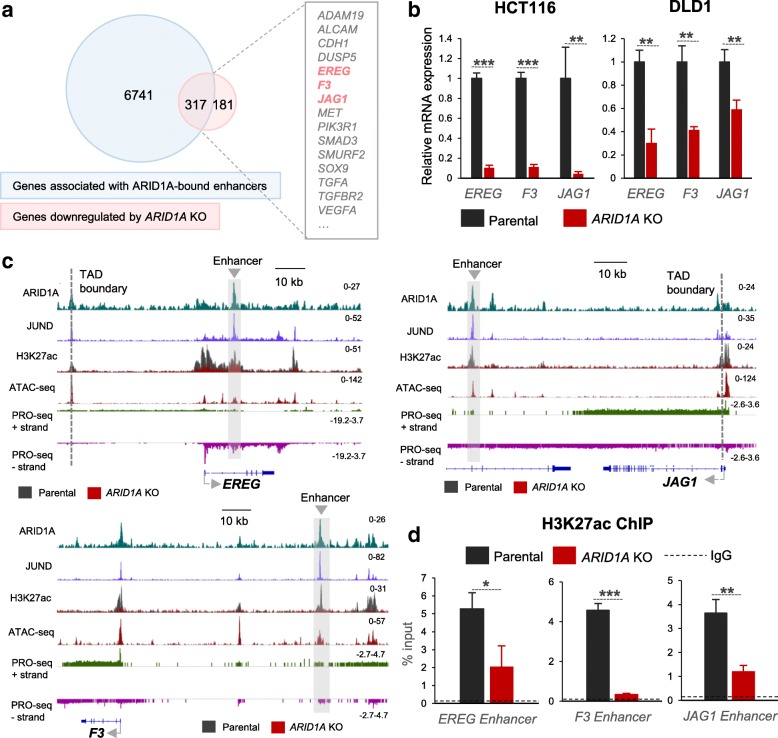
The loss of ARID1A leads to a loss of enhancer activity. Six thousand seven hundred forty-one genes associated with ARID1A-bound enhancers were determined. Three hundred seventeen genes were also downregulated by the knockout of *ARID1A*. Among these were the *EREG*, *F3*, and *JAG1* genes (**a**). The three MEK/ERK pathway target genes identified were downregulated by the deletion of *ARID1A* in both *KRAS*-mutated cell lines (HCT116 and DLD1) (**b**). At the genomic loci for these genes, we identified potential enhancers which are occupied by ARID1A, JUND, and H3K27ac within the same topologically associated domains (TAD). Vertical dotted lines represent TAD boundaries. The potential enhancers are also accessible as assessed by ATAC-seq and are transcriptionally active as assessed by PRO-seq (**c**). There is a significant reduction of H3K27ac at these enhancers upon the deletion of *ARID1A* (**c**, **d**). qRT-PCRs for gene expression and ChIP were run in biological triplicates and technical duplicates. The dotted lines represent the average ChIP-qPCR signal for the negative control IgG. Error bars represent the standard deviation between three biological replicates. Significance was calculated using unpaired *t* test, *p < 0.05, **p < 0.01, ***p < 0.001

After identifying potential ARID1A-bound enhancers and confirming that they are indeed co-occupied by the AP1 factors, we utilized available Hi-C and H3K27ac occupancy data from HCT116 cells to identify potential target genes based on their activity (i.e., H3K27ac-enriched TSS) and presence in the same topology-associated domain. Using this approach, we identified 6,741 genes associated with the 3,061 potential enhancers. Further overlap of these genes with those downregulated following *ARID1A* deletion in HCT116 cells yielded 317 potential direct enhancer targets, including several interesting cancer-relevant genes (Fig. 5a). Interestingly, the *EREG*, *F3*, and *JAG1* genes, which we had identified as being highly dependent on the MEK/ERK pathway, were also identified as ARID1A-bound enhancer targets. Consistently, as shown before, these genes were downregulated by the knockout of *ARID1A* in both *KRAS*-mutated cell lines HCT116 and DLD1 (Fig. 5b). The ChIP-seq tracks shown in Fig. 5c confirm that the ARID1A-occupied enhancer regions on all three genes were also occupied by the AP1 transcription factor JUND and marked by H3K27ac. Importantly, these potential enhancer-promoter pairs are located within the same topologically associated domain (TAD) as assessed by Hi-C, making their interaction more probable. Moreover, PRO-seq (precision run on sequencing) data revealed nascent RNA transcription from these sites, suggesting that eRNAs might be transcribed from these enhancers. These enhancers were also accessible as assessed by ATAC-seq. Contrary to the prevailing view that ARID1A is essential for the maintenance of open chromatin, the loss of ARID1A did not lead to a decrease in accessibility at ARID1A-bound enhancers in HCT116 cells (Fig. 5c, Additional file 1: Figure S2c). While this is surprising, another recent study also reported no reduction of accessibility at enhancers upon the loss of ARID1A in RMG1 cells [46]. Nevertheless, there is a striking loss of H3K27ac from these enhancers, which could partially explain the loss of target gene expression. We confirmed the H3K27ac reduction by ChIP-qPCR at these enhancers (Fig. 5d) and also observed a similar reduction at all ARID1A-bound enhancers (Additional file 1: Figure 2c). Thus, our results show that the loss of ARID1A leads to the reduction of activity of enhancers and target gene expression at regions co-occupied by AP1 transcription factors.

### Attenuation of the MEK/ERK pathway leads to a reduction of ARID1A and H3K27ac at enhancers

**Fig. 6:**
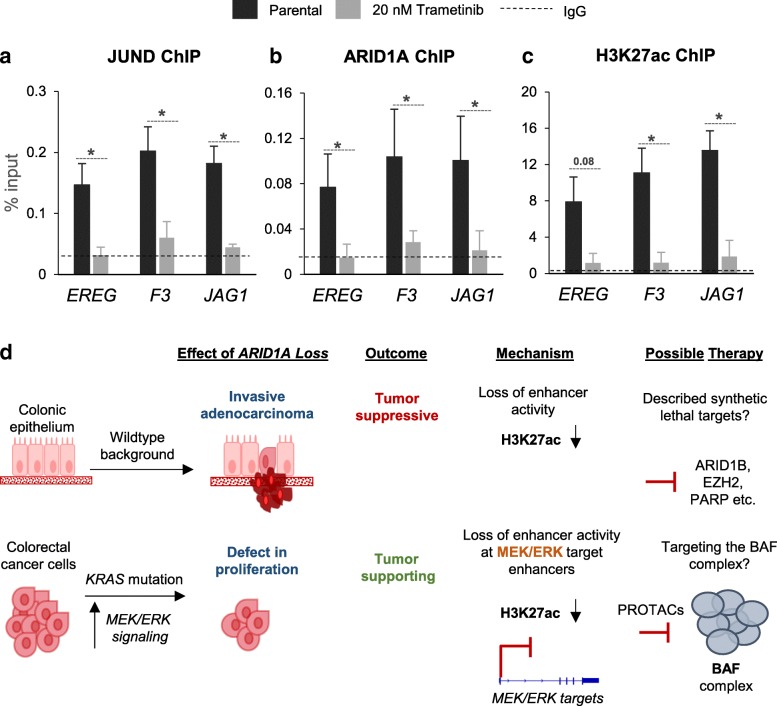
The attenuation of the MEK/ERK pathway leads to a loss of enhancer activity at ARID1A-bound enhancers. Upon treatment with 20 nM trametinib for 24 h, the occupancy of JUND was reduced at the potential enhancers identified (**a**). Moreover, the same treatment also leads to the reduction of ARID1A occupancy and a loss of the H3K27ac mark on these enhancers (**b**, **c**). ChIP was carried out in biological triplicates and qRT-PCRs were run in technical duplicates. The dotted lines represent the average ChIP-qPCR signal for the negative control IgG. Error bars represent the standard deviation between three biological replicates. Significance was calculated using unpaired *t* test, *p < 0.05, **p < 0.01, ***p < 0.001. In summary, we show that the loss of ARID1A has context-dependent functions in colorectal cancer. Mathur et al. describe that in a wild-type background, the loss of ARID1A is tumor suppressive and leads to the formation of invasive adenocarcinomas. This function is mediated through control of enhancer activity wherein there is a loss of the active H3K27ac mark upon the loss of ARID1A [21]. We show that in colorectal cancer cells which harbor *KRAS* mutations, ARID1A has a tumor-supporting function by facilitating transcription at enhancers downstream of the MEK/ERK pathway also partially through the placement of the H3K27ac mark (**d**). In the first case, synthetic lethal targets described in the literature may provide potential therapeutic targets [22, 26, 51–54]. In the case described here, targeting the BAF complex itself using PROTACS could be an option

To further confirm the link between the MEK/ERK signaling pathway and the loss of activity of enhancers, we attenuated the pathway using the clinical MEK1/2 inhibitor trametinib. ChIP-qPCR for JUND at these enhancers confirmed that the MEK/ERK pathway is essential for the maintenance of JUND AP1 transcription factor occupancy at these enhancers (Fig. 6a). Moreover, consistent with our model, treatment with trametinib also resulted in reduced occupancy of ARID1A and H3K27ac at these enhancers (Fig. 6b, c). Thus, inhibition of AP1 transcription factor activity by blocking the upstream MEK/ERK pathway effectively leads to reduced ARID1A recruitment and decreased histone acetylation at ARID1A/AP1-bound enhancers, leading to decreased target gene expression.

## Discussion

The importance of epigenetic regulators in cancer has been widely recognized, and much of the current research is focused on deciphering the role of these regulators in driving oncogenic programs in cancer cells. As described in the previous sections, subunits of the BAF complex are among the most frequently mutated genes in cancer and are extensively described as tumor suppressors. In this study, we explored the role of ARID1A in transcriptional control of gene expression in colorectal cancer cells. We found that cell lines containing activating *KRAS* mutations are particularly dependent on ARID1A. In the absence of ARID1A, the proliferation of these cells is severely impaired, suggesting a tumor-supporting function in this context. Furthermore, we confirmed that ARID1A itself is, indeed, mainly localized at enhancers in colorectal cancer cells where it acts as a co-factor at regions bound by AP1 transcription factors, which act downstream of the MEK/ERK pathway. Consistently, we further showed that the attenuation of the MEK/ERK pathway leads to a disruption of this transcriptional network at enhancers as both ARID1A and H3K27ac are lost from these sites. This is accompanied by a downregulation in the expression of the associated target genes.

At the molecular level, ARID1A acts as a cofactor at sites occupied by AP1 transcription factors. Consistent with our findings, a direct interaction between ARID1A and FOS AP1 factors was previously reported [28]. Our work suggests that this interaction is especially relevant in *KRAS*-mutated colorectal cancers, where an attenuation of the MEK/ERK pathway mimics the effects of ARID1A loss in these cells. Surprisingly, the primary function of ARID1A at these regions does not appear to be dependent upon the ability of the BAF complex to remodel chromatin since its loss had no effect on chromatin accessibility. This may be partially explained by other BAF complexes containing orthologues of ARID1A which may enable chromatin remodeling function [47], but not be sufficient for enhancer activation, or due to the binding of other transcription factors that help maintain chromatin in an accessible conformation. Instead, the ARID1A-containing BAF complexes appear to facilitate the assembly of a larger coactivator complex essential for the activation of enhancers (for instance, through recruitment of a histone acetyltransferase-containing complex). This effect is consistent with what has been reported in *Smarcb1-*deleted mouse embryonic fibroblasts, where H3K27ac is also globally lost from enhancers. Re-expression of *Smarcb1* in that system restored H3K27ac, p300, BRD4, and Mediator binding in the chromatin fraction, supporting that enhancer activity could be restored [32]. In addition to its requirement for the placement of H3K27ac, as a large multi-subunit complex, ARID1A might also facilitate enhancer activity by promoting chromatin looping between the promoter and enhancer. As a complex with many subunits, it appears likely that BAF may play a role in the aggregation of cofactors at enhancers, thereby forming locally active transcriptional hubs.

Under the notion that *ARID1A* is a tumor suppressor, several research groups sought to model *Arid1a*-deficient cancer in mice. While these models confirmed its tumor suppressive role in various cancers, some striking observations were made which indicated that ARID1A could also play a pro-tumorigenic role in certain contexts. For example, in ovarian epithelial cancer (OEC), while *Pten/Apc* deletion led to the formation of poorly differentiated OECs in mice, the additional deletion of *Arid1a* caused tumor cells to have a less aggressive, more differentiated epithelial phenotype [48]. Similarly, deletion of *Arid1a* from otherwise wild-type mice protects against DEN (diethylnitrosamine)-induced hepatocellular carcinoma (HCC), while overexpression of *Arid1a* accelerated tumor initiation [49]. In a pancreatic cancer model, *Arid1a* was shown to have very context-dependent roles [23]. In colorectal cancer, the deletion of *Arid1a* in the background of *Apc* inactivation (which hyperactivates the Wnt pathway) blocked tumor formation [21]. Similarly, deletion of the BAF component *Smarca4* also attenuated Wnt-dependent tumorigenesis [50]. Interestingly, we also observed a strong co-localization of ARID1A with the downstream effector of the Wnt pathway, TCF7L2 (Fig. 4 a), which could explain the cooperation between these factors at the transcriptional level. Thus, the consequences of ARID1A loss in cancer appear to be particularly context-dependent. We show that, in the background of *KRAS* mutation, the loss of ARID1A has an inhibitory effect on proliferation. This finding is further confirmed by the fact that mutations in *KRAS* and *ARID1A* are largely mutually exclusive in colorectal cancer patients. In future studies, it will be very interesting to decipher at which time point during transformation cancer cells lose ARID1A expression. In colorectal cancer, it appears that loss of ARID1A in wild-type backgrounds may promote early tumorigenesis while loss of ARID1A in the background of mutations in *APC* or *KRAS* may inhibit tumor progression (Fig. 6d).

Overall, these findings indicate that the context in which *ARID1A* loss occurs likely determines whether it plays a primarily tumor suppressive or tumor promoting role. Several BAF complex subunits have been described as tumor suppressors in different contexts, making it difficult to use them as direct therapeutic targets. Therefore, several studies have successfully sought to identify synthetic lethal therapeutic targets and identified vulnerabilities that can, at least in principle, be targeted using small molecule inhibitors. While these have shown some promise [22, 26, 51–54], it has become increasingly clear that the context of the tumor must be considered carefully before applying these therapies to ARID1A-deficient cancers. In the case of oncogenic functions, such as in synovial sarcoma or in colorectal cancer as described here, directly targeting the BAF complex could be an option. This could possibly be mediated using PROTACS (Proteolysis Targeting Chimera), which lead to targeted degradation of a cellular protein. Consistently, a PROTAC targeting the BRD9 subunit of the BAF complex has already shown promise in models of synovial sarcoma [55]. Thus, the development of additional PROTACs mediating the degradation of the BAF complex may provide new therapeutic approaches for *KRAS-*mutated colorectal cancer. This would enable a precision oncology approach for the therapy of a relevant fraction of colorectal cancers based on robust molecular mechanisms.

## Conclusions

Taken together, our work significantly expands the knowledge about the context-dependent functions of ARID1A in cancer. We identified that ARID1A is essential in specific contexts of colorectal cancers and was able to show mechanistically that *KRAS-*mutated colorectal cancer cells are especially dependent on the presence of ARID1A. In cells containing *KRAS* mutations, the loss of ARID1A led to decreased activity of enhancers bound by ARID1A and the AP1 transcription factors, and deregulation of target gene expression. This effect was accompanied by impaired proliferation of these cells. Moreover, we also showed that ARID1A has roles beyond its chromatin remodeling activity and can serve as a transcriptional cofactor to promote histone acetylation. This work builds upon and expands existing knowledge about the interplay of ARID1A and AP1 transcription factors by showing its relevance in the development of a subset of colorectal cancers. We also suggest the possibility of the BAF complex being a targetable entity in these cancers. Further exploration of this concept might provide additional important insights into the mechanisms by which ARID1A-containing BAF complexes regulate enhancers and serve as a knowledge base that can be exploited for therapeutic benefit.

## Methods

### Patient data and immunohistochemistry

These experiments were performed according to the guidelines of the local ethics committee and with the requisite informed consent from all patients. Treatment naïve rectal cancer biopsies from 182 patients who were treated at the Department of General and Visceral Surgery, University Medical Center Göttingen, Germany, were collected and tissue microarrays were prepared. Immunohistochemistry for ARID1A was performed on these tissues. The slides were de-paraffinized, rehydrated, and then incubated for 6 min in a pressure cooker (Pascal, Dako) with EDTA and Tween 20 pH 8. After cooling down, the slides were washed and incubated in 3% H_2_O_2_ for 10 min. After washing the slides again, these were incubated with ARID1A antibody (Cell Signaling, 1:500) overnight at 4 °C. The next day, the slides were incubated with the boost-HRP secondary antibody (Cell Signaling) and stained using 3,3′-diaminobenzidine (DAB) (Medac) for 8 min at RT. Counterstaining was performed with hematoxylin. The slides were imaged using an Axioscope microscope (Carl Zeiss). The staining intensities of 164 biopsies were scored by a board-certified pathologist (P.S.).

### Cell culture

HCT116 and HT29 cells were cultured in McCoy’s 5A medium (Gibco), and DLD1 and COLO320DM cells were cultured in RPMI 1640 (Gibco). All cells were tested to be mycoplasma free. All media were supplemented with 10% FBS, 1% glutamine, and 100 U/mL penicillin/streptomycin. Cells were maintained at 37 °C and 5% CO_2_.

For proliferation assays, 5000–7500 cells were seeded in 24-well plates in multiple replicates. Cell proliferation was assessed by measuring confluence using a Celigo S Cell Imaging Cytometer (Nexcelom) every 24 h for 5 days, and plotted as relative confluence over time. The confluence in each well on each day was normalized with the confluence in that well on day one.

For treatment with trametinib (Biomol GmbH), 20 nM of inhibitor was prepared in DMSO and diluted in media. As control, cells were treated with DMSO at a 1:1000 dilution.

### Crystal violet staining

Different conditions were set up in a 24-well format, and cells were grown for 5–7 days. Once confluent, the wells were washed with PBS, fixed in 99% methanol for 10 min, and stained with 1% crystal violet prepared in 2% ethanol.

### Genotyping PCR

Each PCR reaction was conducted in a volume of 25 μL containing 10X buffer B (NEB), dNTPs (Jena Bioscience GmbH), MgCl_2_ (NEB), forward and reverse primers (Sigma), and Taq polymerase (Solis Biodyne). The reaction allowed 15 min of initial denaturation followed by 35 cycles of 30 s at 95 °C for denaturation, 45 s at 60 °C for annealing, and 60 s at 72 °C for elongation. Final elongation was carried out at 72 °C for 10 min. The PCR products were run on a 1% agarose gel at 100 V for 45 min and visualized using an INTAS imager.

### Quantitative real-time PCR (qRT-PCR)

Total RNA was isolated in triplicate using the QIAzol**®** lysis reagent (Qiagen) according to the manufacturer’s instructions. One microgram of RNA was used for reverse transcription by MuMLV reverse transcriptase (NEB) to prepare cDNA. Quantitative real-time PCR was carried out using SYBR Green I (Roche Diagnostics GmbH) as a fluorophore with the following conditions: initial denaturation at 95 °C for 2 min followed by 40 cycles of denaturation at 95 °C for 10 s and annealing and extension at 60 °C for 30 s. qPCRs were analyzed using the standard curve method. For each gene, the expression was normalized to the expression of GAPDH [56]. Primers were designed using NCBI primerblast, and primer sequences are indicated in Additional file 2: Table S2.

qPCR primers for ChIP were designed for the indicated enhancer regions. The following conditions were then used: initial denaturation at 95 °C for 2 min followed by 45 cycles of denaturation at 95 °C for 10 s and annealing and extension at 60 °C for 30 s. Primer sequences are indicated in Additional file 2: Table S2.

### Western blot

Cells were lysed in RIPA lysis buffer (0.5% sodium deoxycholate (*w*/*v*), 0.1% SDS (*w*/*v*), 1% NP-40 (*v*/*v*) containing protease inhibitors). The proteins were denatured by adding Laemmli buffer (375 mM Tris/HCl, 10% SDS, 30% glycerol, 0.02% bromophenol blue, 9.3% DTT) and heating at 95 °C for 5 min. These were then resolved by SDS-PAGE and transferred to nitrocellulose membranes, incubated with primary antibodies (Additional file 2: Table S3) overnight at 4 °C and then in secondary antibodies (Additional file 2: Table S3) for 1 h at RT. The blots were developed using a BioRad ChemiDoc™ imager.

### CRISPR/Cas9-mediated knockout of *ARID1A*

CRISPR/Cas9-mediated gene editing was used to generate *ARID1A-*knockout colorectal cancer cell lines to mimic ARID1A-deficient cancer cells. Four cell lines, namely HT29, HCT116, DLD1, and COLO320DM, were used. Guide RNAs targeting the two introns flanking exon 5 of the *ARID1A* gene were designed, and off-target binding effects were minimized on the basis of scores obtained on the MIT CRISPR design software (http://crispr.mit.edu/). The deletion of the flanked exon (which contained a number of nucleotides not divisible by 3) was predicted to lead to a frameshift in any resulting spliced transcript, thereby leading to the loss of protein expression. The guide RNAs were cloned into a pSpCas9(BB)-2A-GFP (PX458) plasmid which was a gift from Feng Zhang (Addgene plasmid # 48138; http://n2t.net/addgene:48138; RRID: Addgene_48138) [75]. Plasmid DNA (4 μg) was transfected into the cells by electroporation using a Lonza Nucleofector according to the manufacturer’s instructions. Cells were sorted 48 h after transfection as 192 single cell clones by fluorescence-activated cell sorting based on GFP-positivity. The single cell clones were expanded and selected for *ARID1A* knockout. A single clone for each cell line was used for all experiments. Further information on the plasmid and gRNAs is provided in Additional file 2: Table S4.

### Chromatin immunoprecipitation (ChIP)

ARID1A ChIP-seq was performed using a dual-crosslinking approach as described [45]. Briefly, confluent HCT116 cells in 15 cm plates were crosslinked for 20 min in 15 mM EGS (Thermo Fisher Scientific), 20 min in 2 mM EGS at RT, and then in 1% paraformaldehyde (Electron Microscopy Sciences) for 40 min at 4 °C. The samples were then processed using the Active Motif ChIP-IT High Sensitivity Chromatin Immunoprecipitation kit. The samples were sonicated in a Bioruptor Pico (Diagenode) for 15 cycles (30 s on/off) to obtain 200–500 bp fragments. Ten micrograms of sheared chromatin were used for immunoprecipitation overnight with 5 μL of ARID1A antibody (Additional file 2: Table S3, Cell Signaling) at 4 °C. The immunoglobulin complexes were isolated using protein G agarose beads. The beads were washed, and the chromatin was de-crosslinked in the presence of proteinase K at 65 °C overnight. DNA was extracted according to the manufacturer’s instructions.

For H3K27ac and JUND ChIP, confluent cells in 15-cm plates were crosslinked for 20 min by adding 1% formaldehyde (Sigma). For ARID1A ChIP-qPCR, confluent cells in 15-cm plates were crosslinked in 2 mM EGS (Thermo Fischer Scientific) for 40 min at RT followed by crosslinking with 1% paraformaldehyde (Electron Microscopy Sciences) at 4 °C for 40 min. The samples were processed further as follows. Glycine (125 mM) was added to quench the formaldehyde/paraformaldehyde. The cells were harvested in nuclear preparation buffer (150 mM NaCl, 20 mM EDTA pH 8.0, 50 mM Tris-HCl pH 7.5, 0.5% NP-40, 1% Triton X-100, 20 mM NaF, PIC). The nuclear pellet was isolated and resuspended in 150–300 μL sonication buffer-1 (50 mM Tris-HCl pH 8.0, 10 mM EDTA, 1% SDS (*w*/*v*), PIC). After allowing lysis, the SDS content was diluted to 0.5% SDS using 150–300 μL sonication buffer-2 (20 mM EDTA, 50 mM Tris-HCl pH 8.0, 150 mM NaCl, 1% NP-40 (*v*/*v*), 20 nM NaF). The samples were sonicated in a Bioruptor Pico (Diagenode) for 15 cycles (30 s on/off). The soluble chromatin was pre-cleared with unconjugated sepharose. The chromatin was then diluted in dilution buffer (20 mM EDTA, pH 8.0 50 mM Tris-HCl, 150 mM NaCl, 1% (*v*/*v*) NP-40, 20 mMNaF 0.5% (*w*/*v*), sodium deoxycholate) and incubated overnight with primary antibodies (Additional file 2: Table S3). Immunoglobulin-bound complexes were precipitated by adding Protein A sepharose. The beads were washed, the chromatin was de-crosslinked at 65 °C overnight, and DNA was isolated by phenol-chloroform extraction.

### RNA-seq and ChIP-seq library preparation and sequencing

RNA integrity was confirmed by agarose gel electrophoresis. Libraries for poly(A) mRNA-sequencing were prepared using the Capture and Amplification by Tailing and Switching (CATS) RNA-seq kit (Diagenode) according to the manufacturer’s protocol using 50 ng total RNA as the starting material. For ChIP-seq libraries, the Diagenode MicroPlex Library Preparation Kit v2 was used according to the manufacturer’s instructions. Fifty bp single-end mRNA-sequencing was performed by Diagenode, Seraing, Belgium, on a HiSeq 2500. ChIP-seq libraries were sequenced by the Transcriptome and Genome Analysis Laboratory (TAL) of the University of Göttingen on the HiSeq 4000 (50 bp single-end). The concentrations of the libraries were measured using the Qubit 2.0 fluorimeter (Invitrogen), and fragment size was assessed using a Bioanalyzer (Agilent).

### Bioinformatic analysis

#### mRNA-seq data processing

Fastq files for newly generated data have been deposited in ArrayExpress under the accession number E-MTAB-7791. Publicly available raw data (for *ARID1A*-deficient HCT116 cells) was downloaded from the NCBI GEO database (accession numbers are provided in Additional file 2: Table S1). The quality of the sequencing was determined using FASTQC. These were trimmed using specific trimming conditions suggested by the CATS manual. The trimmed fastq files were mapped to the hg19 version of the human genome with Bowtie2 (v 2.2.5) under the TopHat module using the --very sensitive end-to-end setting [57]. The abundances of the various transcripts in the obtained BAM files were estimated by CuffLinks, and differential expression analysis between different conditions was carried out using CuffDiff [58]. For further analysis in differential expression, those genes which showed *q* value ≤ 0.05, log2FC ≥ 0.7, or ≤ − 0.7 for HCT116 and log2FC ≥ 0.6 or ≤ − 0.6 for DLD1 and COLO320DM were used.

#### ChIP-seq and ATAC-seq data processing

Fastq files were obtained from the Transcriptome and Genome Analysis Laboratory (TAL) of the University Medical Center Göttingen (the data is deposited in ArrayExpress under the accession number E-MTAB-7792). Publicly available raw data were downloaded from NCBI GEO database (accession numbers are provided in Additional file 2: Table S1). The quality of the sequencing was determined using FASTQC. The first 12–13 bp from the 5′ end were trimmed using FASTX trimmer where necessary. The trimmed fastq files were mapped to the hg19 version of the human genome with Bowtie 2 (v 2.2.5) using the --very sensitive end-to-end setting [57]. The BAM files obtained were sorted and indexed using SAMTOOLS [59]. In the ATAC-seq datasets, mitochondrial reads were removed. These files were converted to BigWig format using BamCoverage [60] and visualized using the Integrated Genome Viewer (IGV) tool [61]. Coverage was normalized to 1X setting, and an extension length of 200 bp was used (for ChIP-seq datasets, ATAC-seq data was paired-end and was not extended). Peaks were called using the MACS2 software [62], and BED files were obtained. The --nomodel setting was used, and the FDR *q* value to call peaks was set at ≤ 0.05. Broad peaks (cutoff of 0.05) were called for histone modifications and narrow peaks for transcription factors and ATAC-seq data.

#### Functional analysis and integration of ChIP-seq and RNA-seq data

The GeneVenn online tool was used to create Venn diagrams of gene lists. Processed RNA-seq data was subjected to Gene Set Enrichment Analysis (GSEA) [63, 64]. Gene Ontology (GO) and pathway enrichment analysis were performed using the EnrichR software [65, 66] using lists of downregulated genes as input. HOMER (v 4.8) was used to find motifs enriched in ChIP-seq datasets [67]. Scrambled sequences of the input file were used as background. The Genomic Regions Enrichment of Annotations Tool (GREAT) was used to find region associations [68]. Associated genes were determined by using a custom-made algorithm considering TAD boundaries from Hi-C data as well as gene activity based on H3K27ac ChIP-seq data. The ReMap analysis tool was used to find co-localizing transcriptional regulators using a BED file as input [69]. Heatmaps and aggregate plots were created using the reference point mode of the computeMatrix deepTools tool followed by plotProfile or plotHeatmap [60]. Profiles and heatmaps were plotted relative to the center of the peaks provided in the input BED file ± 5 kb.

#### Hi-C and PRO-seq data processing

Fastq files were downloaded from NCBI GEO (accession numbers in Additional file 2: Table S1). The Hi-C data was analyzed using HiCExplorer [70]. Briefly, reads were mapped against hg19 with bwa [71], and then a matrix was built with 2 kb resolution. Subsequently, corrections of the matrix were done to remove bias of GC, open-chromatin, and number of restriction sites within a certain bin. The corrected matrix was then used to call TADs (topologically associated domains).

The PRO-seq data was analyzed as previously described [72]. The reads were first mapped against hg19 with bowtie2 [57]. The low-quality mapped reads (MQ < 30) were discarded, and the duplicates were removed. BAM files were then converted to bedGraph format, and only the 3′ ends of fragments were kept to achieve single-base resolution. Next, bedGraph files were converted to bigwig files for visualization of nascent transcription.

### Statistical analyses and graphs

Graphs and tables for Figs. 1a and 3e and Additional file 1: Figure S1b were generated from the cBioPortal for Cancer Genomics [3, 4]. For calculating statistical significance to compare parameters between different conditions, the unpaired *t* test was used. *P* values were determined by this test and significance depicted as ***p ≤ 0.001, **p ≤ 0.01, *p ≤ 0.05. Statistical tests for the analysis of NGS data were performed by the indicated software.

## Additional files

Additional file 1: **Figure S1.** Expression of BAF complex subunits in the four cell lines used in this study (a). Mutual exclusivity of *ARID1A* and *KRAS* (all and specifically at residues G12 and G13) mutations in the colorectal adenocarcinoma patient cohort from the TCGA PanCancer Atlas (b). Levels of pERK and JUND in Parental and *ARID1A* KO DLD1 cells (c). HSC70 was used as a loading control. The top 10 GO terms enriched for genes downregulated by *ARID1A* KO in the HCT116, DLD1, and COLO320 cell lines (d). **Figure S2.** Transcription factors that colocalize at all ARID1A-bound sites. These include several AP1 transcription factors (a). The AP1 binding motif is significantly enriched at all ARID1A-occupied regions (b). At all ARID1A-bound enhancers there is a reduction of SMARCA4 and SMARCC1 occupancy upon the loss of ARID1A (c). ATAC-seq signal remains unchanged and H3K27ac reduces significantly (c). (PDF 126 kb)

Additional file 2: Supplementary tables. This file contains supplementary **Tables S1–S4.** (DOCX 31 kb)

## Data Availability

The datasets generated and during the current study are available in the ArrayExpress repository, https://www.ebi.ac.uk/arrayexpress/experiments/E-MTAB-7791, https://www.ebi.ac.uk/arrayexpress/experiments/E-MTAB-7792 under accession numbers E-MTAB-7791 (m-RNA-seq) and E-MTAB-7792 (ChIP-seq). Publicly available datasets accessed are available in the following: NCBI GEO: SMARCC1, SMARCA4, H3K27ac ChIP-seq https://www.ncbi.nlm.nih.gov/geo/query/acc.cgi?acc=GSE71510 [21] NCBI GEO: FOSL1, JUND ChIP-seq https://www.ncbi.nlm.nih.gov/geo/query/acc.cgi?acc=GSE32465 [73] NCBI GEO: ATAC-seq https://www.ncbi.nlm.nih.gov/geo/query/acc.cgi?acc=GSE101966 [47] NCBI GEO: HCT116 RNA-seq https://www.ncbi.nlm.nih.gov/geo/query/acc.cgi?acc=GSE71511 [21] NCBI GEO: Hi-C and PRO-seq https://www.ncbi.nlm.nih.gov/geo/query/acc.cgi?acc=GSE104333 [74]
