# Supplementary material for: ARID1A facilitates KRAS signaling-regulated enhancer activity in an AP1-dependent manner in colorectal cancer cells
Source: Clin Epigenetics. 2019 Jun 19;11:92. doi: 10.1186/s13148-019-0690-5 (PMC6585056; doi:10.1186/s13148-019-0690-5)

Supplemental Fig. 1

a)

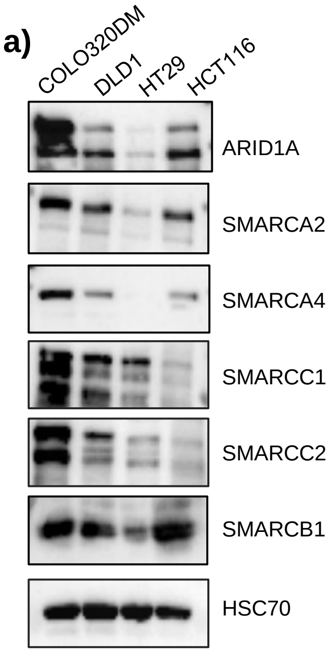

b) Mutual Exclusivity of *ARID1A* and *KRAS* mutations in the TCGA PanCancer Atlas Colorectal Adenocarcinoma Cohort (526 patients)

Number of patients with *ARID1A, KRAS*, both or neither mutations

ARID1A	*KRAS*	Both	Neither	Log2 Odds Ratio	p-value	Mutual Exclusivity
44	204	16	262	-1.098	0.007	Mutually exclusive

ARID1A	*KRAS* *	Both	Neither	Log2 Odds Ratio	p-value	Mutual Exclusivity
49	168	11	298	-1.328	0.004	Mutually exclusive

* KRAS mutations at residues G12 and G13

c)

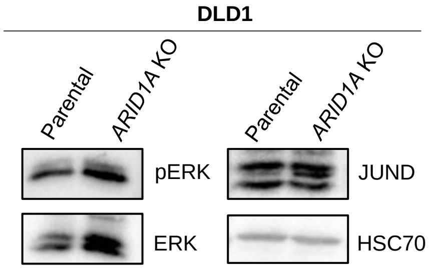

d) **Top 10 pathways enriched in *ARID1A* KO downrgulated genes**

HCT116	DLD1	COLO320DM
**HCT116 *ARID1A* KO downregulated GO Terms**	**DLD1 *ARID1A* KO downregulated GO Terms**	**COLO320DM *ARID1A* KO downregulated GO Terms**
regulation of transcription from RNA polymerase II promoter	positive regulation of chemotaxis.	positive regulation of cardiac muscle cell differentiation.
response to endoplasmic reticulum stress	regulation of chemotaxis.	positive regulation of cardiocyte differentiation.
positive regulation of transcription from RNA polymerase II promoter	actin filament bundle assembly.	regulation of cardiac muscle cell differentiation.
epidermal growth factor receptor signaling pathway	actin filament bundle organization.	hair cell differentiation.
hippo signaling	platelet degranulation.	positive regulation of cell differentiation.
positive regulation of transcription, DNA-templated	regulation of actin cytoskeleton organization.	cGMP-mediated signaling.
intrinsic apoptotic signaling pathway	extracellular matrix organization.	regulation of relaxation of cardiac muscle.
intrinsic apoptotic signaling pathway in response to endoplasmic reticulum stress.	positive regulation of locomotion.	regulation of Wnt signaling pathway, planar cell polarity pathway.
regulation of epithelial cell proliferation.	negative regulation of tumor necrosis factor-mediated signaling pathway.	regulation of leukocyte degranulation.
regulation of fat cell differentiation.	regulation of cytoskeleton organization.	cAMP catabolic process.

Supplemental Fig. 2

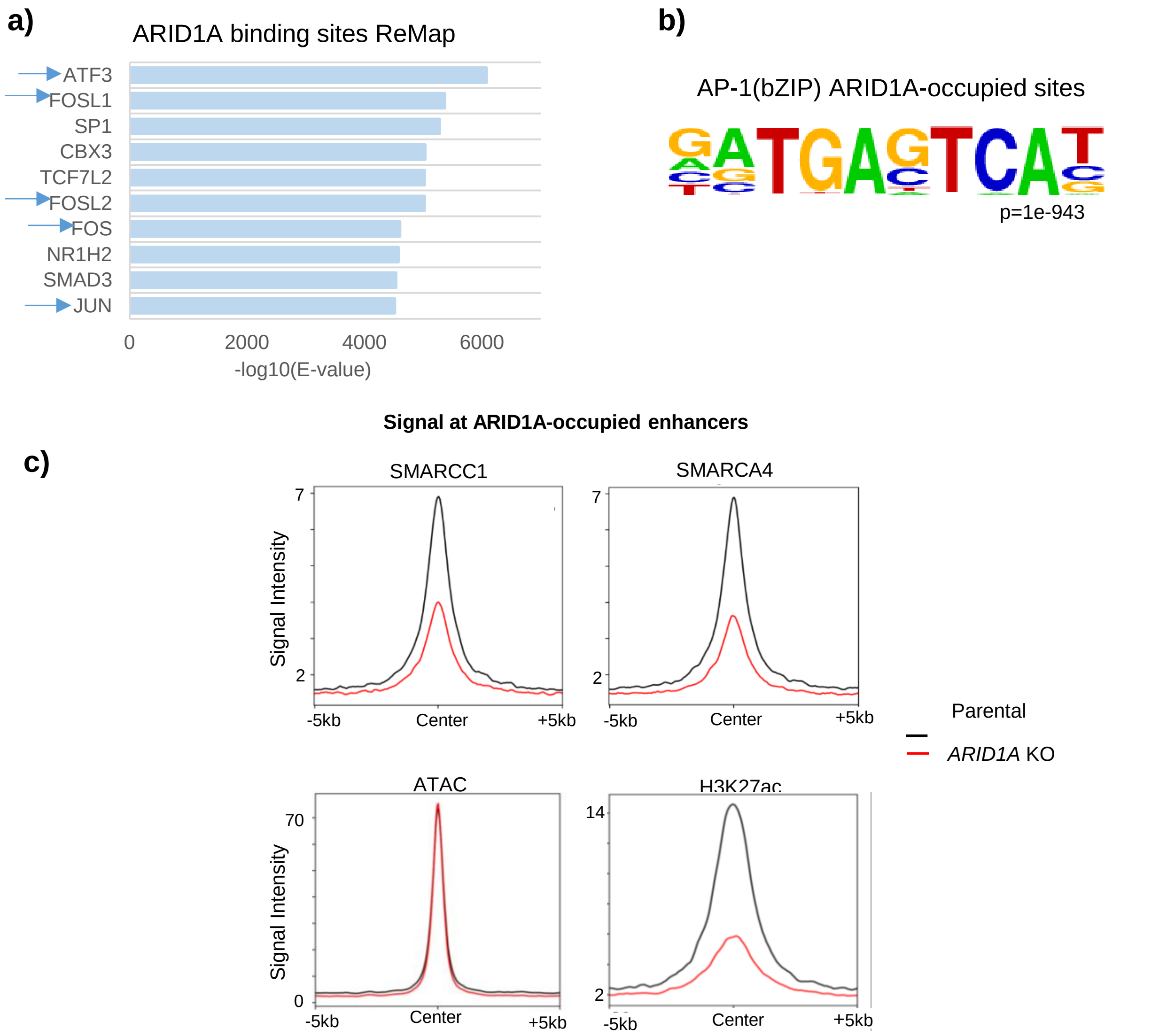

Supplement: Supplementary file 1 — Figure S1. Expression of BAF complex subunits in the four cell lines used in this study (a). Mutual exclusivity of ARID1A and KRAS (all and specifically at residues G12 and G13) mutations in the colorectal adenocarcinoma patient cohort from the TCGA PanCancer Atlas (b). Levels of pERK and JUND in Parental and ARID1A KO DLD1 cells (c). HSC70 was used as a loading control. The top 10 GO terms enriched for genes downregulated by ARID1A KO in the HCT116, DLD1, and COLO320 cell lines (d). Figure S2. Transcription factors that colocalize at all ARID1A-bound sites. These include several AP1 transcription factors (a). The AP1 binding motif is significantly enriched at all ARID1A-occupied regions (b). At all ARID1A-bound enhancers there is a reduction of SMARCA4 and SMARCC1 occupancy upon the loss of ARID1A (c). ATAC-seq signal remains unchanged and H3K27ac reduces significantly (c). (PDF 126 kb) [file 13148_2019_690_MOESM1_ESM.pdf]
