# Supplementary material for: ARID1A facilitates KRAS signaling-regulated enhancer activity in an AP1-dependent manner in colorectal cancer cells
Source: Clin Epigenetics. 2019 Jun 19;11:92. doi: 10.1186/s13148-019-0690-5 (PMC6585056; doi:10.1186/s13148-019-0690-5)
Supplement: Supplementary file 2 — Supplementary tables. This file contains supplementary Tables S1–S4. (DOCX 31 kb) [file 13148_2019_690_MOESM2_ESM.docx]

**Supplemental Figure Legends**

**Supplemental Figure 1:** Expression of BAF complex subunits in the four cell lines used in this study (a). Mutual exclusivity of *ARID1A* and *KRAS* (all and specifically at residues G12 and G13) mutations in the Colorectal Adenocarcinoma patient cohort from the TCGA PanCancer Atlas (b). Levels of pERK and JUND in Parental and *ARID1A* KO DLD1 cells (c). HSC70 was used as a loading control. The top 10 GO terms enriched for genes downregulated by *ARID1A* KO in the HCT116, DLD1 and COLO320 cell lines (d).

**Supplemental Figure 2:** Transcription factors that colocalize at all ARID1A-bound sites. These include several AP1 transcription factors (a). The AP1 binding motif is significantly enriched at all ARID1A-occupied regions (b). At all ARID1A-bound enhancers there is a reduction of SMARCA4 and SMARCC1 occupancy upon the loss of ARID1A (c). ATAC-seq signal remains unchanged and H3K27ac reduces significantly (c).

**Supplemental Table 1**

**Accession numbers for publicly available NGS datasets used**

| **ChIP-seq and ATAC-seq** | **GEO Accession** | **Reference** |
| --- | --- | --- |
| SMARCC1 (WT and *ARID1A* KO)  SMARCA4 (WT and *ARID1A* KO)  H3K27ac (WT and *ARID1A* KO) | Series GSE71510 | Mathur et al., 2017 [21] |
| ATAC-seq (WT and ARID1A KO) | Series GSE101966 | Kelso at al., 2017 [47] |
| FOSL1 | Series GSE32465 | ENCODE [73] |
| JUND | Series GSE32465 | ENCODE [73] |
| **RNA-seq** |  |  |
| WT and ARID1A KO | Series GSE71511 | Mathur et al., 2017 [21] |
| **Hi-C and PRO-seq** |  |  |
| HCT116-RAD21-mAC | Series GSE104333 | Rao et al., 2017 [74] |

**Supplemental Table 2**

**Primer Sequences**

**Genotypying PCR**

| **Gene** | **Forward Primer (5’-3’)** | **Reverse Primer (5’-3’)** | **Reference** |
| --- | --- | --- | --- |
| ARID1A knockout | ACTGGAAGAAGACAAAAGTGC | CTGCTGCTCCAGACAAAGAA | This study |

**Gene Expression qRT-PCR**

| **Gene** | **Forward Primer (5’-3’)** | **Reverse Primer (3’-5’)** | **Reference** |
| --- | --- | --- | --- |
| EREG | CTCTGCCTGGGTTTCCATCTTC | TCACTGGACTCTCCTGGGATAC | This study |
| F3 | ACCTGGAGACAAACCTCGGA | TCCCGGAGGCTTAGGAAAGT | This study |
| GAPDH | ATGGGGAAGGTGAAGGTCG | GGGGTCATTGATGGCAACAATA | [56] |
| JAG1 | ACCCCCTGTGAAGTGATTGAC | CTGACTCTTGCACTTCCCGT | This study |

**ChIP qRT-PCR**

| **Gene** | **Forward Primer (5’-3’)** | **Reverse Primer (3’-5’)** | **Reference** |
| --- | --- | --- | --- |
| EREG_enh_TF | AGCAAGGTCAAAATAAACCGTATGC | AAGTGGTTGCCCAACAGTCA | This study |
| EREG_enh_ac | ATCATCTGTGTTATATCACCTGGCA | TTTCTTGTCTGGTGGCATTGGT | This study |
| F3_enh_TF | CACCGACGAGATTGTGAAGGA | CCGAGGTTTGTCTCCAGGTAAG | This study |
| F3_enh_ac | TGCTTCCGAGTTGGCTGTAG | CTCAGCCCACTAACCGTCTTT | This study |
| JAG1_enh_TF | TGCCCTAGATAGAGAAGGGATGAA | AATCGCAAACTTTCGGACACAC | This study |
| JAG1_enh_ac | GATACGCCTTCGCTGCATCA | CGCAAACTTTCGGACACACTC | This study |

**Supplemental Table 3**

**Primary antibodies**

| **Antibody** | **Species** | **Clone** | **Source** | | **Cat. No.** | **Dilution** | |
| --- | --- | --- | --- | --- | --- | --- | --- |
|  | | | | | | **WB** | **ChIP** |
| ARID1A | Rabbit | D4A8U | | CST | 12354 | 1:1000 | 5µL |
| ERK | Rabbit | Polyclonal | | Santa Cruz | sc-94 | 1:1000 | - |
| JUND | Rabbit | Polyclonal | | Santa Cruz | sc-74 | 1:1000 | 1.5µg |
| H3K27ac | Rabbit | Polyclonal | | Diagenode | c15910196 | - | 2µg |
| HSC70 | Mouse | B-6 | | Santa Cruz | sc-7298 | 1:30000 | - |
| IgG | Rabbit | Polyclonal | | Abcam | ab46540 | - | 1.5-5µg |
| pERK | Rabbit | Polyclonal | | CST | 9101 | 1:500 | - |
| SMARCA2 | Rabbit | D9E8BXP^®^ | | CST | 11966 | 1:1000 | - |
| SMARCA4 | Rabbit | A52 | | CST | 3508 | 1:1000 | - |
| SMARCB1 | Rabbit | Polyclonal | | Diagenode | C15410317 | 1:1000 | - |
| SMARCC1 | Rabbit | D7F8S | | CST | 11956 | 1:1000 | - |
| SMARCC2 | Rabbit | D8O9V | | CST | 12760 | 1:1000 | - |

**Secondary antibodies**

| **Antibody** | **Cat. No.** | **Dilution** | **Source** |
| --- | --- | --- | --- |
| Goat Anti-Mouse IgG-HRP | [sc-2004](http://www.scbt.com/datasheet-2004-goat-anti-rabbit-igg-hrp.html) | 1:10,000 | Santa Cruz |
| Goat Anti-Rabbit IgG-HRP | [sc-200](http://www.scbt.com/datasheet-2004-goat-anti-rabbit-igg-hrp.html)5 | 1:10,000 | Santa Cruz |
| SignalStain (R) Boost IHC Detection Reagent (HRP, Rabbit) | 8114S |  | Cell Signaling |

**Supplemental Table 4**

**Information on CRISPR/Cas9 experiments**

| Vector name | pSpCas9(BB)-2A-GFP (PX458) | Addgene #48138  Reference: [75] |
| --- | --- | --- |
| Bacterial resistance | Ampicillin | |
| Promoter | hU6 | |
| Name of gRNA | hARID1A-3 | hARID1A-4 |
| Sequence of insert (sense) | CACCGCAGTGTTTCACTCGTTGCC | CACCGGACTGCCCCCAGTAATATTA |
| Sequence of insert (antisense) | AAACGGGCAACGAGTGAAACACTGC | AAACTAATATTACTGGGGGCAGTCC |
| Targeted Gene | hARID1A-Ex5-A | hARID1A-Ex5-B |
| NCBI reference sequence | NM_006015.4 | |
| Resulting modification | Knock out | |
| Restriction enzyme | BbsI (BpiI) | |
